# Increasing Household Diet Diversity and Food Security in Rural Rwanda Using Small-Scale Nutrition-Sensitive Agriculture: A Community-Engaged Proof-of-Concept Study

**DOI:** 10.3390/nu15143137

**Published:** 2023-07-14

**Authors:** Brittney C. Sly, Tiffany L. Weir, Leslie Cunningham-Sabo, Stephen J. Leisz, Valerie J. Stull, Christopher L. Melby

**Affiliations:** 1 Department of Food Science and Human Nutrition, Colorado State University, Fort Collins, CO 80523, USA; tiffany.weir@colostate.edu (T.L.W.); leslie.cunningham-sabo@colostate.edu (L.C.-S.); chris.melby@colostate.edu (C.L.M.); 2 Department of Anthropology and Geography, Colorado State University, Fort Collins, CO 80521, USA; steve.leisz@colostate.edu; 3 Center for Sustainability and the Global Environment, University of Wisconsin Madison, Madison, WI 53706, USA; vstull@wisc.edu

**Keywords:** diet diversity, nutrition-sensitive agriculture, food security, kitchen gardens, participatory action research

## Abstract

Malnutrition and food insecurity remain high in rural Rwanda, where residents consume a low-diversity diet provided through subsistence farming. Agricultural interventions using kitchen gardens may improve diet diversity in some populations. However, little is known about their efficacy when developed using community-based participatory research in combination with nutrition education focused on the empowerment of women. The objective of this study was to develop and implement a kitchen garden and nutrition education intervention using a community-engaged model and examine its impact on household diet diversity and food security. Using a mixed methods community-level design, we assessed a 16-week intervention implemented in Cyanika, Rwanda. Stratified purposeful sampling was used to select women participants representing 42 households. Household diet diversity scores (HHDS) and hunger scores were calculated at the baseline, post-intervention and one-year follow-up. HDDS increased after intervention from a pre-intervention intake of 2.59 [1.3] food groups/day, to 4.85 [1.6] at four months post-intervention and at one year post-intervention, reaching 5.55 [1.3]. There were no significant changes in household hunger scores. Our results indicate that collaborative community-engaged nutrition-sensitive agricultural interventions can increase household diet diversity; however, future work should explore whether this type of intervention strategy can lead to sustained changes and impact nutritional adequacy in this population.

## 1. Introduction

In 1994, Rwanda experienced a devastating genocide that decimated the population, infrastructure, and economy. Since then, the country has made admirable progress in rebuilding its economy and infrastructure, while improving living standards for its population of 12.5 million people. However, a large proportion of the population still faces pervasive poverty, malnutrition, and food insecurity. Recent surveys indicate that 39.1% of the population lives in poverty, mostly concentrated in rural areas, where 83% reside [1]. A majority of people (70%) engage in the agricultural sector for income, supplying 90% of the country’s food needs, with most practicing subsistence farming [1]. Despite national efforts targeted at rural residents, improvement in rates of malnutrition, particularly among women and children, have stagnated; this is largely attributed to a lack of gains in household food security and inadequate diet consumption [2,3]. In 2021, Rwanda reported 20.6% of households remained food insecure, predominantly in rural areas where agriculture is the main income source [3]. Additionally, 27% of households were consuming an inadequate diet, meaning the variety of foods consumed as determined over a seven-day period was less than optimal to ensure sufficient nutritional balance [3]. These data corroborate the estimated 48% of children age 6–59 months who are vitamin A deficient, and the 32.4% of children under the age of 5 who are classified as stunted, with the greatest proportion represented by children living in rural areas [3,4]. Approximately 13% of women aged 15–49 years of age are anemic, with a higher prevalence among pregnant women living in poorer households [5]. In the Burera District, a rural area located in the Northern Province of Rwanda, nutrition-related indicators show a more severe situation with approximately 43.1% of households reporting food insecurity and 45–60% reporting inadequate food consumption, based on food consumption scores [3]. The prevalence of childhood stunting (41%) is higher than the national rate (32.3%) [3].

Despite most rural residents engaging in agriculture, prior work with this population indicates a general lack of knowledge regarding small-scale vegetable production, nutritious food choices, and healthy food behaviors, particularly regarding diet diversity. Traditionally, the female head of the household prepares daily meals from available local food, gathered from cash crop stores or local markets, resulting in a primarily starch-based, low-variety diet. Small-scale fruit and vegetable production via kitchen gardens has been a somewhat effective solution for addressing these issues in other populations [6,7,8,9,10]. According to a 2016 review by Pandey et al., of the 25 studies examined, interventions that increased household crop diversity and livestock ownership also increased household-level diet diversity, child dietary diversity, and the consumption of nutrient-dense animal foods, fruit and vegetables, and micronutrient intake [11]. Another review concluded that projects providing agricultural resources, while also investing in human capital through nutrition education and gender considerations, had the greatest likelihood of effecting positive changes in nutrition [12]. Specifically, small-scale fruit and vegetable production via kitchen garden projects were identified as nutrition-sensitive agriculture interventions having the highest success rate due to their ease of adoptability, investment in human capital, and women’s empowerment aspects [12]. A better understanding of traditional gender roles, and how they intersect with agricultural production and health-related behaviors, would supply researchers with valuable insight to adjust research strategies more appropriately. Thus, a tailored approach that allows the identification and incorporation of these influencing factors into intervention strategies could help to improve the sustainability of intervention outcomes.

Despite the evidence that women’s empowerment is a key strategy, there is no consensus on the best application for empowerment methods that can be adopted to multiple cultural and community contexts. Thus, there are limited data on how to implement women’s empowerment strategies. One approach is to apply a method of research that allows the culture and community to be a part of the solution. Participatory action research (PAR) is an innovative approach that seeks to understand and improve the world through collective, self-reflective inquiry undertaken by researchers and participants together, thus leading to action [13]. Directly linked through action, and influenced by the understanding of culture and social context, engaging in the process of research is empowering for community participants, thus leading people to have increased control over their lives [14].

One of the most common techniques for PAR research is the use of *PAR Cycles* that apply structure and organization to the constant reflection, analysis, and action that embodies PAR. Generally, several cycles are performed during a project and coordinate with the many evaluations and phases that occur. Another common technique, embodied as participatory rural appraisal, combines traditional in-depth anthropological evaluation methods, such as large questionnaire surveys or in-depth anthropological methods including semi-structured interviews, with transect observation walks, mapping and diagramming, all conducted by the people within the targeted community [15,16]. Ultimately, this approach has led to more inclusive methods of program evaluation that better address international development projects by enabling local people to share, add to, and analyze their knowledge of the intended subject within the context of their individual lives and communities—leading to a plan and action for change [16]. By coupling nutrition-sensitive agriculture interventions with the empowering and inclusive methods adapted from PAR, this model can provide and evaluate a framework for establishing site-specific and sustainable nutrition-sensitive agriculture interventions.

Using PAR approaches and peer-training methods, an exploratory intervention model was developed. The model aimed to educate and empower rural Rwandan women by expanding on their existing agricultural and nutrition knowledge with the primary goal of increasing household food security and diet diversity through kitchen gardening. This study assessed if the developed intervention was successful in (1) enhancing household dietary diversity by increasing availability of a variety of home-grown fruits and vegetables, and (2) improving overall household food security by providing consistent food access via kitchen gardens. It was hypothesized that this intervention would result in changes in household dietary diversity consistent with increased food security, which could equate to improved diet quality. Sustainable increases in these parameters could remediate and prevent malnutrition. Finally, this research could provide a model for collaborative food security interventions to be used as a basis for program design and evaluation with similar populations and communities that face malnutrition and food insecurity at a regional and global scale.

## 2. Materials and Methods

This study was conducted in a rural community in the Northern Province of Rwanda. The research was conducted in collaboration with a U.S.-based non-governmental organization (NGO) that has worked within the community since 2006. Prior to the start of the study, a comprehensive exploratory assessment was conducted with a sample of the intended study population. To ensure culturally appropriate community entry, numerous meetings were held to discuss the intervention and work with community leaders, co-establish the intended methods of the study, and determine the intervention topics and materials. Institutional review board approval was obtained from Colorado State University (ID: 19-9040H), and all study participants provided written informed consent.

### 2.1. Study Design and Subjects

Study participants were recruited from the Cyanika area located in the Burera District in northern Rwanda. Participants were chosen based on the location of their permanent residence within the district to enhance geographic diversity, their perceived need for assistance, absence of a kitchen garden at their residence, and their willingness to train others in the future. Each participant was then placed in a group for the duration of the study. Forty-two non-pregnant women >18 years of age and considered the female head of household were selected by community liaisons using a stratified purposeful sampling method. Women were selected for this exploratory intervention study because of their traditional role as decision makers regarding food for the household. Past research has identified women as an ideal conduit for malnutrition remediation interventions in rural poor agriculture-based populations throughout sub-Saharan Africa [9,17,18]. Purposeful sampling provided a limited number of cases, facilitating the acquisition of in-depth analysis that could guide the investigators to understand the central problem under study [19]. This method was appropriate because of the homogeneity of the groups and the research goal of examining variation in key outcomes [20]. Randomized sampling was not feasible as the long-term goal of the intervention was to provide sustained change and generate information spread within specific communities. Therefore, allowing community liaisons, who were respected members of the community, to recruit and choose participants based upon the study criteria, led to greater community buy-in, which was essential to the success of the intervention. Participants were recruited from each of the six government-established geographically specific community groups, referred to as *cells*. Each cell contained seven participants, and a leader for each cell was chosen internally by the group. The initial forty-two participants were referred to as project *Ambassadors* to foster empowerment and emphasize their future role as trainers.

### 2.2. Intervention

Prior to the start of the study, a comprehensive exploratory assessment was conducted with a sample of the intended study population in collaboration with the Cyanika Community Vision Board, partnering NGO leaders, and local coordinators. The assessment provided insight to researchers and community leaders about the intended methods of the study, parameters for participants, and development of the intervention materials.

From January to May 2019, each cell group participated in a 16-week intervention that included lecture-based training, hands-on farming activities, and socialization. Members of each cell met weekly for approximately 2 h in their separate cell groups at the cell leader’s home, known as the *Demonstration Site*. Project coordinators and subject-matter community experts, such as a *Community Nutrition Health Worker* and an *Agronomist*, led the training sessions. The time commitment was kept to what was considered reasonable by participants, but still provided them ample return on their commitment through increased knowledge and access to their home-grown vegetable crops. Materials and curriculum specific to this study were developed to educate participants and provide specific learning topics on a weekly basis (Table 1).

Participants created keyhole-style and raised-bed gardens at their homes, as well as compost piles and rainwater catchment systems for irrigation. They also learned about how to prepare balanced meals for all members of their household using the fruits and vegetables they grew in their kitchen garden. Resources were provided throughout the intervention, including workbooks and notepads, gardening tools, seeds, and construction materials. Some resources were intended for personal use, and others were intended to be shared. Due to the varied education and literacy levels, materials primarily included pictures and illustrations. During the final week of the intervention, a large group session was conducted with all forty-two Ambassadors using PAR methods to guide self-reflection and knowledge assessment, while encouraging empowerment of the women participants. Three PAR activities were conducted by U.S. researchers and local project coordinators with all attendees—(1) *Participatory Mapping*, (2) *Matrix Scoring*, and (3) *Commitment Writing*. By applying these PAR activities at the end of the intervention, the participants and researchers completed a complete PAR cycle and thus informed the participants and the community about how to continue with the intervention over the long term. This manuscript focuses on the methods and quantitative aspects of the study relating to household diet diversity and hunger outcomes, while the qualitative methods and corresponding data are discussed elsewhere [21].

### 2.3. Data Collection

Data were collected at three time points during the study, selected to capture seasonal variations in food availability: (1) baseline data were collected in November 2018, during a time when this region experiences fairly constant weather patterns with occasional rainfall and mild temperatures; (2) four months post-intervention in September 2019, during what is considered their ‘sunny’ season, characterized by low rainfall, limited crop growth, and historically low food security and diet diversity; and (3) one year post-intervention in June and July 2020, in order to evaluate the sustainability of intervention goals [22].

Semi-structured interviews were used to collect data on diet diversity and food security. The lead researcher (BCS) conducted interviews with the assistance of a local translator. Interview questions and responses were translated in real time from English to Kinyarwanda and back by a trained translator. The translator was a university student and local member of the community, who completed training in research ethics along with conceptual training with the lead researcher by way of a bilingual individual not involved in the study. From these instructional exercises, the research team determined the interviewer and translator to be able to collect accurate data in accordance with the study protocol.

#### 2.3.1. Sociodemographics

Sociodemographic details were collected from interviewer-administrated questionnaires completed during semi-structured interviews. Data were obtained concerning household size and occupants, marital status, sources of income, sources for obtaining food, and whether they engaged in cash-crop agriculture.

#### 2.3.2. Dietary Assessment

Dietary assessment was performed using a 24 h open recall administered by a trained registered dietitian to estimate the ‘usual’ intake of the household at each of the three time points using the woman head of household as the proxy. Participants were asked to include all foods eaten by all household members, including meals, snacks, and foods eaten in and outside the home. Although using multiple 24 h recalls over several days is considered the best reference for assessing diet diversity, evidence from prior research indicate that using a single 24 h recall in rural sub-Saharan African populations is sufficient to predict regular dietary intake as compared to a standard three-day recall [23].

#### 2.3.3. Household Diet Diversity Score

Dietary information gathered from respondents was applied to a diet diversity equation as performed in previous studies with similar populations, calculated by summing the number of different food groups consumed by individuals in a household over the 24 h recall period [9]. Information recorded from the 24 h recalls was used to determine a household diet diversity score (HDDS) based upon adaptation of the Food and Agriculture Organization (FAO) tool to include regional and culture-specific foods [24]. Protein foods are defined as foods from the following groups: *Organ and Flesh Meat*, *Eggs*, *Fish and Seafood*, *Legumes, Nuts and Seeds*, and *Milk and Milk Products*. HDDS is a qualitative measure that reflects household access to a diversity of foods and has been shown to also serve as a proxy for the nutrient adequacy of the diet of individuals in a household [9,25,26]. The HDDS is calculated based on the number of food groups consumed out of twelve possible food groups (Table 2) [25]. A second coder not involved with the collection of the original data independently applied the dietary assessment data from seventeen participants, representing 15% of the total sample, to the diet diversity calculator to check inter-rater reliability. One hundred percent agreement was established between the coders.

#### 2.3.4. Food Security Evaluation

Household food security was evaluated during interviews at all three data collection time points. The validated Household Hunger Scale developed by the Food And Nutrition Technical Assistance (FANTA) Project was utilized given its accuracy depicting comparable indicators across cultures and within food-insecure populations [24]. Measurements of household hunger were used as a proxy for household food security, through a series of questions used to determine a score for each household, with a higher score equating to more hunger or greater food insecurity [27]. Three questions were asked during each interview to tabulate a Household Hunger Score (HHS) that represented a household hunger category [27]. A second coder not involved with the collection of the original data independently calculated the HHS from recorded interview data for seventeen participants, representing 15% of the total sample, to check inter-rater reliability. One hundred percent agreement was established between the coders.

### 2.4. Data Analysis

The Statistical Package for Social Sciences software version 26 (SPSS Inc., Chicago, IL, USA) and GraphPad Prism software version 8 (GraphPad Software, San Diego, CA, USA) were used to conduct all statistical analyses. Descriptive data was depicted as means and standard deviations, or percentages. The percentages of households consuming each of the different food groups according to HDDS were determined. A linear mixed-effects model was used to analyze the changes in HDDS and HHS across all time points depicted as means with 95% confidence intervals. ANOVA using a post hoc Tukey’s test was used to analyze between subject variation, and Pearson’s correlation coefficient was used to determine potential confounders attributing to individual variability in HDDS and HHS, which were then further analyzed using independent *t*-tests. For all statistical analysis, a *p* value of ≤0.05 was accepted as significant.

## 3. Results

### 3.1. Characteristics of Study Groups

Among the forty-two participants representing their respective households (mean [SD] age, 41.9 [12.3] years), the majority were married, and a smaller percentage were widowed, separated, or divorced at the time of the study (Table 3). The average number of people living in each household was six, with 67% of households having at least one child under the age of five years. Like other rural populations in Rwanda, many participants reported growing staple food crops such as potatoes, maize, beans, sorghum, and sweet potatoes that their household consumed and/or sold for income. The main income source for participants was working in agriculture for other local farmers (66.7%), with 11.8% reporting no source of income. A smaller proportion of households obtained income from a household member working as a retailer of goods at local markets, having formal employment, such as being a teacher, hair stylist, pastor, or having other sources of income such as charitable gifts from the church or family members.

### 3.2. Household Dietary Diversity Measurements

**Figure 1:**
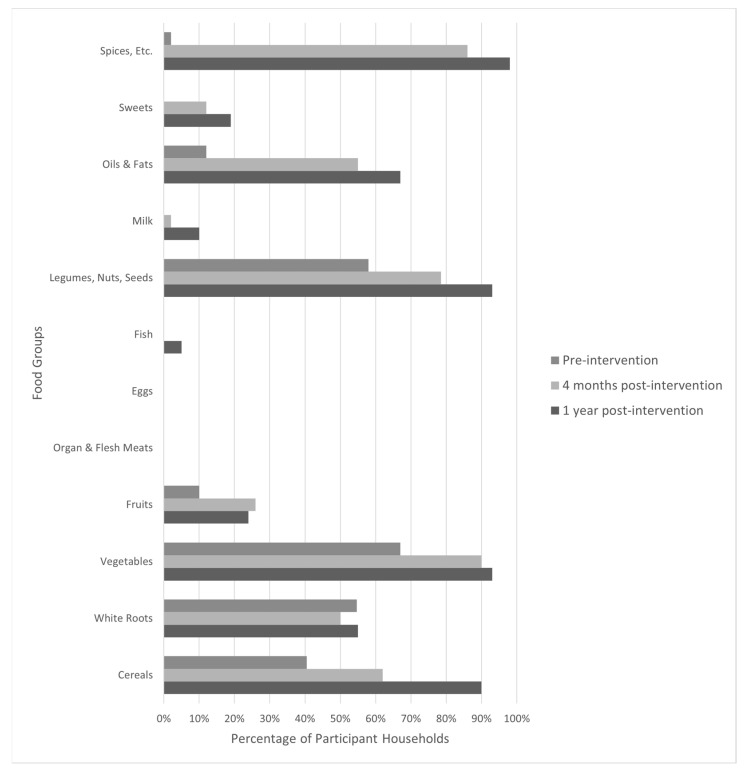
Proportion of participant households consuming foods from each food group during each time point (Cyanika, Rwanda).

At the baseline, the average household diet diversity score (mean [SD]) was reported as 2.6 food groups from the 24 h dietary recall exercises [1.3]. Prior to the intervention, there were three food groups that provided the bulk of the food consumed by most households, including *White Roots* (55%), primarily in the form of Irish potatoes, *Legumes*, *Nuts*, and *Seeds* (58%) in the form of dried beans, and *Vegetables* (67%) in the form of leaves from cassava plants, locally known as *isombe*, or leaves from bean plants (Figure 1). Additionally, at the baseline, 67% of households were consuming foods considered vitamin A rich, and 55% of households were consuming protein foods, as defined in Table 2.

**Figure 2:**
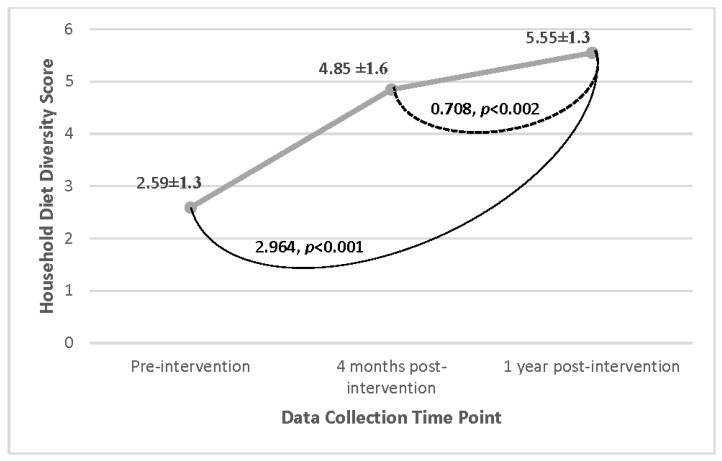
Changes in HDDS over time for all participant households on a scale of 0–12 (Cyanika, Rwanda). Linear mixed effects statistical analysis of the mean household diet diversity scores comparing changes across the different time points.

Amongst all participant households, the average HDDS significantly increased over time (mean [SD] from pre-intervention (2.6 food groups [1.3])) increased to four months post-intervention (4.9 [1.6]) and continued to increase one year post-intervention (5.6 [1.3]) (Figure 2). The average HDDS four months post-intervention indicated households consumed an average of 1.9 more food groups than they did pre-intervention, and 2.9 more food groups one year post-intervention than pre-intervention, showing a consistent increase over time. These data also indicate that during the ‘sunny’ season (four months post-intervention), when the availability of food is historically scarcer leading to inadequate and unbalanced dietary patterns, increased household diet diversity was observed.

During the data collection period four months post-intervention, the number of food groups consumed increased for all food groups, except for foods included in the *Organ and Flesh Meats*, *Eggs*, and *Fish* groups. Data collected one year post-intervention showed a continued increase in the number of all food groups consumed. Most notably, foods in the *Cereals*, *Vegetables, Spices,* and *Legumes*, *Nuts*, and *Seeds* food groups were consumed by over 90% of participants (Figure 1).

**Figure 3:**
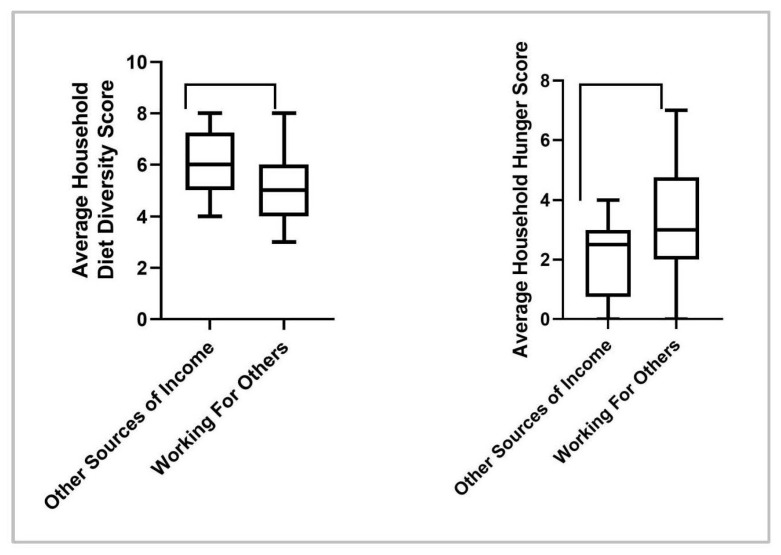
Average household diet diversity scores and household hunger scores for women participants (Cyanika, Rwanda), in relation to the main income source of household. The higher the household hunger score, the more food insecure a household.

Among households whose main source of income came from working for other farmers, HDDS scores were significantly lower [*t*(40) = −2.108, *p* = 0.041] than for those households with other sources of income (Figure 3). In addition, households whose main source of income came from an employed household member reported significantly higher HDDS [*t*(39) = 12.940, *p* < 0.001] than those households with other sources of income.

**Figure 4:**
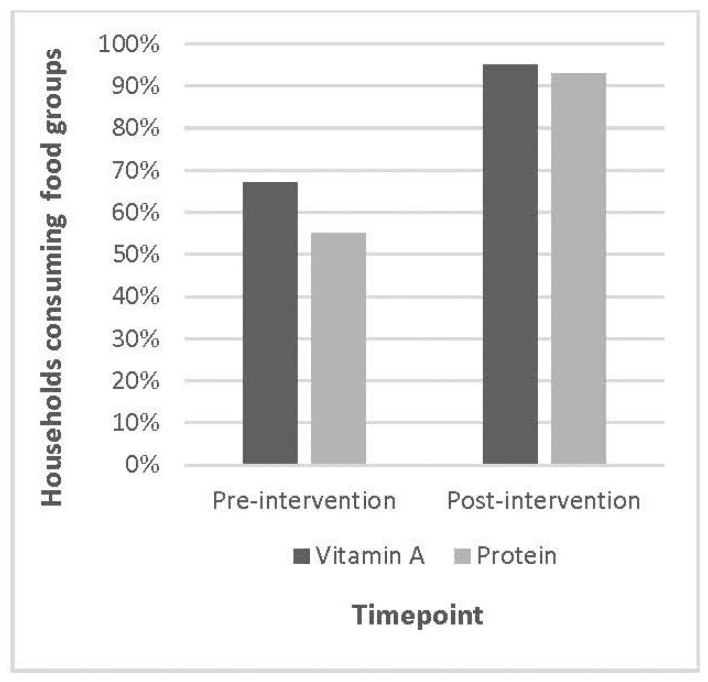
Proportion of participant households consuming vitamin A-rich foods and protein foods pre- and post-intervention (Cyanika, Rwanda).

To determine if the changes in household diet diversity were associated with improved nutritional adequacy, the proportion of participant households consuming vitamin A-rich foods and protein foods (as defined in Table 2) at the three time points was examined. Post-intervention, the proportion of households consuming vitamin A-rich foods increased to 95%, and those households consuming protein foods increased to 93% (Figure 4).

Additionally, among those households that did not report consuming protein foods and vitamin A-rich vegetables at the baseline, all reported consuming foods from these food groups post-intervention. In addition, a large majority (83%) of the participant households growing vitamin A-rich vegetables in their kitchen gardens also consumed these vegetables.

Thirty-one percent of participants who were consuming ‘other’ vegetables were also growing vegetables included in the ‘other’ category. However, 42% of participants who reported growing ‘other’ vegetables did not report their consumption within their respective households. The vegetables grown with the highest frequency in response to the intervention were amaranth leaves (locally known as *dodo*, a dark-green leafy vegetable (57%), onions (55%), green cabbage (52%), beets (50%), and carrots (43%). Note that prior to the intervention, none of the women had kitchen gardens and thus were not growing any of their own vegetables for consumption.

### 3.3. Household Food Security Measurements

**Figure 5:**
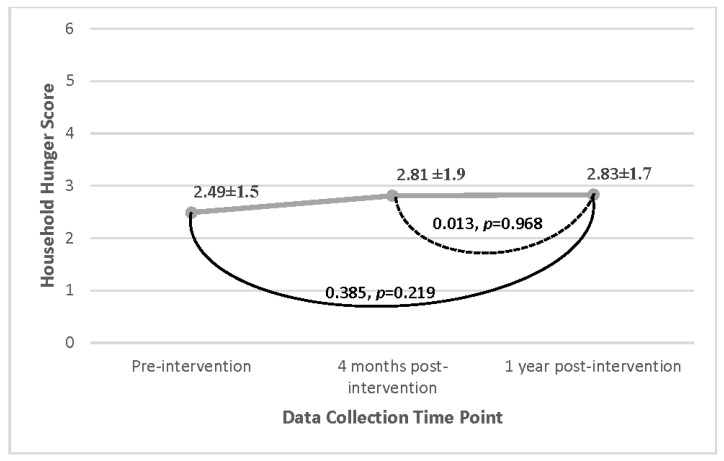
Changes in household hunger scores over time for all participant households on a scale of 0–6 (Cyanika, Rwanda). Linear mixed effects statistical analysis of the mean household hunger score comparing changes across the different time points. Results were non-significant.

At the baseline, the average HHS score (mean [SD]) was reported as 2.49 [1.5] from the semi-structured interviews, indicating moderate hunger levels [27]. The results for household food security are represented in Figure 5. There were no significant changes in HHS scores for any participants across all time points, indicating no change in the level of household food security from pre- to post-intervention. Additionally, despite improvements in post-intervention HDDS described previously, the HHS did not change, showing no relationship between changes in HDDS and observed changes in HHS. It was observed, however, that households whose main source of income was working for other farmers, reported a significantly higher HHS *t*(40) = −2.090, *p* = 0.043, than those households with other sources of income, indicating greater food insecurity in the former. In addition, households whose main source of income came from an employed household member reported significantly lower HHS *t*(40) = −2.017, *p* = 0.05, than those households with other sources of income, indicating lower food insecurity.

## 4. Discussion

Based on previous studies, it was hypothesized that this exploratory nutrition-sensitive kitchen garden intervention would be associated with a sustained increase in household diet diversity leading to better food security, thus indicating an improvement in dietary patterns aimed at reducing malnutrition. The key findings were an increase in average household diet diversity four months after the intervention, during the sunny season, as well as one year after the intervention, suggesting sustained change. However, the intervention did not result in significant improvements in household food security.

Average household diet diversity consistently increased over time, showing sustained change. One year post-intervention, households consumed on average five or more food groups during the 24 h period, out of a possible twelve food groups, with increases in all food groups except those shown in Figure 1. Unlike individual diet diversity measurements, household diet diversity measurements do not have a standard cutoff for food groups that will equal nutritional adequacy. Rather, household diet diversity is used to measure the availability of food, and thus, a discussion of patterns is better suited for these data [28]. The biggest increase was observed in the *Spices*, etc., food group with an increase of over 80%, which could be interpreted as inconsequential as the gardens did not provide spices directly. Included in the intervention, however, were recipes and a group cooking class conducted by a *Nutrition Health Worker* from the community, with the intention of introducing new ways to cook the vegetables grown in their gardens and prepare meals that are nutritionally adequate. Therefore, it is not surprising that an increase in this food group was observed, if only to increase the palatability of meals as introduced during the intervention. Additionally, upon further analysis, iodized table salt was the only spice reported, providing the essential micronutrient iodine, often lacking in the diets of poor rural populations globally [29]. In addition, the inclusion of many nutrient-dense foods increased by at least 20% from the baseline to September 2019 to include foods in the following groups: *Cereals*, *Vegetables*, *Oils and Fats*, *Fruits*, and *Legumes*, *Nuts and Seeds*. Within the *Vegetables* food group, variety and nutritional density increased substantially, particularly with participants consuming more colorful varieties such as spinach, beets, carrots, and onions, providing health-promoting phytochemicals. As stated previously, the percentage of households that included Vitamin A-rich vegetables, such as dark leafy greens and carrots, increased from pre- to post-intervention, according to their 24 h recall (Figure 4). However, since no biomarker data were collected on actual micronutrient levels in participants, no conclusions can be reported on the actual nutrient status of participants or household members—a limitation that could be addressed in future studies. However, the variety of vegetables offered by kitchen gardens likely contributed to household diet diversity both directly and indirectly, which is consistent with other research in similar populations [6,9,10].

The results also indicate that other foods groups, apart from vegetables, either continued to increase or were added one year post-intervention. For example, the number of households that consumed foods in the *Cereals* food group in the form of sorghum, wheat, and maize flours, or meals used for making porridge, all increased from pre- to post-intervention. These foods, grown primarily as cash crops in this region and purchased in the market, are considered some of the more expensive foods. It is possible that households were able to save money from not purchasing vegetables from the market, which afforded them greater opportunities to purchase other items such as cereals, salt, oil, and, in a few cases, milk and fish. Thus, it is possible that kitchen gardens not only increased the consumption of a variety of nutrient-dense vegetables, but also allowed for foods in other food groups to be consumed due to more income flexibility. Increasing income flexibility in a household is an indicator of progress toward poverty reduction, a primary driver of food insecurity, by facilitating the purchase of nutrient-dense foods, rather than just those that meet energy needs [6,30]. Therefore, further research is warranted on the impacts of this interventional approach on income flexibility.

There was considerable variability in the magnitude of changes in household diet diversity among the different participant households. The reasons are unclear but could be associated with differences in the income source of the household among the groups. As reported, households whose main source of income was from working on the farms of others saw lower diet diversity than those households that had someone working in the market or having formal employment (Figure 3). This has also been reported in similar rural populations where those having even a slightly higher, more stable income source was associated with greater diet diversity [6]. It is possible that the informal economy observed in rural areas where agriculture is the main provider contributes to the seasonality and unpredictability of employment, representing a level of instability in this workforce versus those that have a steady and stable income [30]. During the sunny season, many residents of Cyanika report no source of income due to the seasonality of crop production in the area. In accordance with historical data, this would account for the lower diet diversity previously reported during this time [31]. However, as part of the intervention, rainwater collection tanks were distributed, and education on water management techniques was provided to help tackle this barrier. Concurrent with a study by *Taruvinga* et al., who examined barriers to increasing diet diversity in rural households located in South Africa, water management resources may have assisted in the success of the intervention by allowing kitchen gardens to thrive and provide a diverse amount of foods to households through the sunny season [6]. In the future, initiatives focused on innovative water management techniques for small-scale agriculture could greatly influence the success of nutrition-sensitive agriculture interventions as water is a constant barrier for many rural African communities.

The intervention did not result in significant changes in household food security, as shown in Figure 5. Similar correlations with income source and household hunger were reported, as was seen with HDDS, wherein those households whose main source of income came from working on the farms of others saw greater food insecurity than those households that had someone working in the market or having formal employment (Figure 3), suggesting that those with more stable employment also have more consistent access to enough food.

While an explanation for no reported change in overall household food security is not readily apparent, there are a few things to consider. First, due to the nature of the study being exploratory, the shorter timeframe may not be enough to reflect any changes. Second, through previous work with this population and community, the NGO has observed an inclusive and compassionate collective attitude toward others. Following the Rwandan genocide in 1994, the country as a whole adopted stronger, collective cultural values such as unity, selflessness, volunteerism, and humility, which are apparent both in everyday life and in the culture, and they are exhibited by way of many of their government policies [32,33,34]. This compassion is often translated into assisting others who are in need through the giving of money, food, household goods, employment opportunities, and childcare. When considering this in the context of food security, many participants reported that they gave extra food away to others, possibly hindering their own household food security status.

Another potential factor influencing the HHS was insufficient training and resources for food preservation and storage, as identified during qualitative data collection and discussed elsewhere. Food preservation practices and storage capabilities of extra food stores is a difficult barrier to overcome when refrigeration is nonexistent and dry, reliable storage facilities are hard to maintain, as is the case in rural Rwanda. As reported in another study, this research corroborates that this barrier also exists at the household level, where inadequate storage facilities prohibited participants from keeping food for future periods, potentially contributing to the lack of improvement in food security [35]. Therefore, more research is needed to develop ways that households can store their extra food while helping the community at the same time, so as not to degrade existing cultural values. Perhaps the next step for this community would be to consider larger community gardens that resonate with cultural values by potentially supplying more community members with food, thus allowing those with kitchen gardens to store their excess for future use.

Regular border closures with neighboring Uganda, as well as the on-going COVID-19 pandemic, may have also impacted outcomes from the intervention. Cyanika is located 4.6 km from the Ugandan border, where many residents cross daily for work and the trading of goods. As reported by study participants, when the border is closed, the amount of agricultural work available to residents of Cyanika is considerably less, causing many to be without an income for their household. These closures occur quite regularly due to violent and political conflict between the two countries, as well as regional health concerns. During this research, the border was closed several times for weeks to months. In addition, domestic stay-at-home orders due to the COVID-19 pandemic also affected household income. As reported, the source of household income for our participants was associated with variability in diet diversity and food security measures; thus, it can be assumed that the disruption of these sources hindered some of the progress toward more sustained food security. A recent study in Kenya and Uganda reported decreases in diet quality and increased food insecurity during this time, specifically affecting income-poor households, thus corroborating our observations [36]. Although border closures and global health emergencies are not within the scope of control for this research, continued research on farmer resiliency and market integrity that informs policy is essential for the forward progress of nutrition-sensitive agriculture interventions as outlined by global organizations in recent years [18,37].

In addition to previously aforementioned limitations present in this study, there are others that should be noted. First, the collaborative structure of the study within the community could result in response bias, as participants are also members of the community and thus have a stake in the outcomes. This is especially true when the community and/or participants are receiving resources. Second, we used HDDS to evaluate food availability and food access but did not collect the amount of foods consumed or nutrition-related biomarkers, both of which measure nutritional adequacy. Therefore, we cannot reliably predict that the foods consumed were in sufficient amounts to meet nutritional adequacy. Third, the exploratory nature of this study, with a small sample size and no control group, provided results that can only suggest that the intervention had a positive impact on household diet diversity and, thus, subsequent dietary patterns. Although considered as part of the study design, a control group was not used in this study for a few reasons. First, the overall goal of the research design was to enhance the potential of sustainability through collaborative participatory methods and women’s empowerment. Therefore, it was against the participatory nature of the research design to control any information spread throughout the community. Second, including a control group would have been inappropriate owing to ethical questions of assigning some women/families to a non-intervention group, thus limiting their resources despite high rates of poverty and food insecurity. Third, this research was conducted in collaboration with an NGO that had an already-established rapport with the community, lending trust and assurances that aided in the success of the intervention. Having a control group within the community would breakdown the trust and rapport established by the partnering NGO. Last, the collectivist and generous characteristics of Rwandan culture made it difficult to establish a control group due to the high risk of cross-contamination. Therefore, in this exploratory study, we chose to design and conduct the research without a control group, to respect the collaborative nature of the overall project while knowing the limitations. However, in the absence of a control group, the many strengths of this study provide direction for future research and a potential intervention structure that could yield sustained changes to food security through more diverse food access.

## 5. Conclusions

This study demonstrated that a collaborative community-engaged nutrition-sensitive agricultural intervention in a rural Rwandan community was associated with increases in household diet diversity for up to one year post-intervention. Using kitchen gardens as the conduit for change, households can increase their consumption of home-grown vegetables with increasing household income flexibility, leading to the opportunity for purchasing other nutritious foods. The reasons for the lack of improvements in food insecurity in the face of increased dietary diversity are unclear, and they necessitate additional research on systems and structures impacting food availability and agricultural markets.

## Figures and Tables

**Table 1:** Weekly intervention curriculum and learning topics.

Week	Learning Topic
1	Introduction to basic nutrition and home gardens
2	Trench garden design
3	Keyhole garden design
4	Work week
5	Water management and the nutritional needs of various household members (part 1)
6	Composting and different nutrients in food
7	Work week
8	Pest and weed management and the nutritional needs of various household members (part 2)
9	Seed saving and household meal planning
10	Work week
11	Perennial crops, fruit trees, and nutritional deficiencies
12	Food safety and preservation and ensuring household food security
13	Cooking and planning balanced meals
14	Work week
15	Question-and-answer session
16	Large group reflection and celebration

**Table 2:** A list of the 12 food groups and the foods contained within each group according to the FAO, adapted for regional and culture-specific foods. Specific food groups of nutritional interest are bolded.

Food Groups	Foods within the Food Group
**Cereals**	maize, rice, wheat, sorghum, millet, or any other grains or foods made from these (e.g., bread, noodles, ugali)
**White Roots and Tubers**	white potatoes, white yams, or white cassava
**Vegetables**	**Vitamin A-Rich Vegetables and Tubers: pumpkin, carrot, squash, or sweet potatoes that are orange inside, ibihaza, tomato** **Dark-Green Leafy Vegetables: dark-green leafy vegetables including spinach, kale and wild forms and/or locally available vitamin A-rich leaves such as from amaranth (dodo), cassava (isombe)** Other Vegetables: any other vegetables (e.g., tomato, onion, eggplant, cabbage, onion, lettuce, celery)
**Fruits**	**Vitamin A-Rich Fruits: mango, cantaloupe, apricot, papaya, peach, and 100% fruit juice made from these fruits** Other Fruits: matoke (banana variety), passion fruit, avocado, plantain, pineapple, and other fruits, including 100% fruit juices made from these fruits
**Organ and Flesh Meats**	**liver, kidney, heart, or other organ meats, and beef, pork, lamb, goat, rabbit, chicken, duck, other birds, insects, and wild game**
**Eggs**	**eggs from chicken, duck, or guinea fowl**
**Fish and Seafood**	**fresh or dried fish and seafood**
**Legumes, Nuts, and Seeds**	**dried beans, dried peans, lentils, nuts, seeds, or foods made from these foods (e.g., peanut butter, ikinyiga)**
**Milk and Milk Products**	**milk, cheese, yogurt, or other milk products**
**Oils and Fats**	oil, butter, margarine added to food or used for cooking
**Sweets**	sugar, honey, sweetened beverages, sugary foods such as candies, cookies, cakes, sweet bread foods
**Spices, Condiments, and Beverages**	spices (salt and black pepper), condiments (ketchup sauce, mayonnaise), coffee, tea, alcoholic beverages

**Table 3:** Participant characteristics, Cyanika, Rwanda; summer 2020 (*n* = 42).

Age (Years), Mean (SD)	41.9 (12.3)
Marital status, percentage	
Married	81.0%
Widowed	16.7%
Separated/Divorced	2.4%
Number of people in household, mean (SD)	6.0 (2.2)
Number of children in household under the age of 5, mean (SD)	1.0 (0.9)
Grow staple crops (potatoes, maize, beans, sorghum and sweet potatoes), percentage	62%
Main income source, percentage	
Retailer	11.9%
Work for Other Farmers	66.7%
Employed	4.8%
Other	4.8%
No Income Source	11.8%

## Data Availability

The datasets used and/or analyzed during the current study are available from the corresponding author on reasonable request.
